# Progression-Free Survival Efficacy in Refractory/Relapsed Multiple Myeloma among Elderly Patients: A Systematic Review

**DOI:** 10.3390/life13122259

**Published:** 2023-11-27

**Authors:** Tung-Lung Yang, Chin Lin, Ching-Liang Ho, Tzu-Chuan Huang, Yi-Ying Wu, Hong-Jie Jhou, Po-Huang Chen, Cho-Hao Lee

**Affiliations:** 1 Division of Hematology and Oncology Medicine, Department of Internal Medicine, Tri-Service General Hospital, National Defense Medical Center, Taipei 114, Taiwan; doc10897@mail.ndmctsgh.edu.tw (T.-L.Y.);; 2 School of Public Health, National Defense Medical Center, Taipei 114, Taiwan; 3 Department of Research and Development, National Defense Medical Center, Taipei 114, Taiwan; 4 Department of Neurology, Changhua Christian Hospital, Changhua 500, Taiwan; 182902@cch.org.tw

**Keywords:** frail, elderly, refractory and relapsed multiple myeloma

## Abstract

Background: Over the last decade, many studies have assessed the efficacy of treatments for refractory/relapsed multiple myeloma (R/R MM). While combination therapies show greater efficacy than traditional methods, limited research has targeted elderly patients who might be less resilient to treatments. Our study aimed to evaluate treatment efficacy for these elderly patients. Methods: We carried out a comprehensive review of the literature using a systematic approach. Initially, 4966 citations were retrieved and subsequently narrowed down to 13 eligible randomized controlled trials (RCTs) through our systematic review process from databases like Embase, PubMed, and Cochrane Library from 1 January 2000 to 31 December 2022. Evidence was collated through a frequentist network meta-analysis, using the hazard ratio (HR) for evaluation. Results: Combined therapy of daratumumab, lenalidomide, and dexamethasone (DaraLenDex) was the preferred treatment for R/R MM elderly patients. Its strengths included an HR for progression-free survival (0.15; 95% CI: 0.09–0.25) and a 96% P-score. Conclusions: Our analysis suggests that, pending more comprehensive RCTs, DaraLenDex is the treatment with the highest efficacy for R/R MM in elderly patients.

## 1. Introduction

Multiple myeloma (MM) is a form of cancer originating from plasma cells and is characterized by the production of monoclonal immunoglobulin [1]. It ranks as the second most common hematological malignancy, accounting for 1% of all types of cancer and 2% of all cancer deaths [2,3]. MM predominantly impacts older individuals, with an average diagnosis age of around 70 years, with approximately two-thirds of patients being over 65 years old [4,5].

However, treating MM in this population can be challenging due to several factors. Elderly patients often have comorbidities and pre-existing organ dysfunction, which may compromise treatment outcomes. Additionally, chemotherapy-related adverse events are more common in elderly patients and have a higher risk of chemotherapy toxicity. Frailty scores tend to be poorer in elderly patients, and their goals of care can vary, necessitating a highly personalized treatment approach. They may also be more vulnerable to medication interactions and adverse events, particularly from the novel agents that are becoming increasingly incorporated into MM treatment regimens.

The current standard treatment for fit myeloma patients under 65 years is proteasome inhibitors (PIs) combined with immunomodulatory imide drugs (IMiDs) followed by high-dose therapy (HDT) plus autologous stem cell transplantation (ASCT). Recent advancements, including the introduction of newer agents like carfilzomib, lenalidomide, and pomalidomide, have significantly enhanced the response rate and prognosis for younger MM patients [6,7]. However, elderly patients who are older than 65 years old and unsuitable for HDT–ASCT have different treatment options.

The incorporation of PIs and IMiDs alongside standard chemotherapy has transformed the treatment approach for both initial and refractory/relapsed (R/R) multiple myeloma patients, leading to significant extensions in both progression-free survival (PFS) and overall survival (OS) [8]. However, the elderly population exhibits significant heterogeneity, encompassing individuals who are fit, intermediate-fit, and frail. Elderly impairment is prevalent in elderly patients, especially those with comorbidities, which may determine whether elderly patients can complete treatment or not. Particularly for aged patients who failed first-line therapy, the ability to tolerate multiple lines of treatment for relapse becomes more difficult [9].

One of the major concerns in treating the elderly population is premature discontinuation due to toxicity, which may compromise efficacy and decrease quality of life while active disease is still present. The challenge is to provide efficacy and tolerable treatment options that consider the unique needs of elderly patients with MM while avoiding excessive toxicity and maintaining a good quality of life. In recent years, monoclonal antibodies have emerged as a promising therapeutic option for elderly patients with R/R MM, and daratumumab, in particular, has demonstrated significant efficacy in clinical trials.

Several clinical trials have demonstrated that daratumumab-based regimens have significant efficacy in terms of improving PFS and overall response rates in the elderly population with R/R MM. Additionally, daratumumab has a better safety profile, with a low risk of treatment-related adverse events. Given their potential efficacy and favorable safety profile, daratumumab-based regimens should be further investigated in future clinical trials as a treatment option for elderly MM patients.

Thus, a network meta-analysis (NMA) of RCTs comparing the treatment efficacy for the elderly R/R MM population is necessary and of interest. This would allow for a comprehensive analysis of the existing data and offer clinicians more evidence-based treatment options for this challenging patient population.

## 2. Materials and Methods

To perform the current network meta-analysis, we established guidelines based on the Preferred Reporting Items for Systematic Reviews and Meta-Analyses extension for Network Meta-Analyses (PRISMA-NMA, Table S1) [10,11]. The protocol for the systematic review and meta-analysis can be found at the following register address: https://osf.io/hqkj4/ (accessed on 1 December 2022).

### 2.1. Search Strategy

We searched for relevant publications electronically using databases such as Pub-Med, Embase, the Cochrane Collaboration database, and proceedings from major international meetings in hematology and oncology. Prospective studies were considered for inclusion in this analysis only [12].

To identify all relevant trials, we used comprehensive search strategies, and all titles and abstracts were screened. (Search strategies and detailed records are shown in Scheme S1). In addition, we performed gray literature searches and manually reviewed articles referenced in review articles that could potentially meet the eligibility criteria. We also searched ongoing clinical trials from the US Government Clinical Trials database (www.ClinicalTrials.gov, accessed on 1 December 2022). Citations were excluded for various reasons, such as review, study phase, intervention, disease, study design, meta-analysis, patient population, economic status, and other criteria.

The period covered by our systematic literature search was from 1 January 2000 to 31 December 2022. Included in the analysis were studies that outlined a randomized controlled trial (RCT) involving patients with R/R MM and had subgroup analyses of elderly patients. Additionally, the RCT regimens needed to incorporate at least one of the predefined novel treatments. Following the elimination of duplicates, we assessed citations for eligibility.

### 2.2. Inclusion Criteria

Only studies meeting specific criteria were considered for inclusion in the analysis. They were required to encompass patients diagnosed with R/R MM, including those undergoing second-line or subsequent treatments, be RCTs with or without blinding, or be abstracts or unpublished data with sufficient information on study design, participant characteristics, interventions, and outcomes. The experimental arm of the studies involved patients receiving a nontraditional or new regimen, while the control arm included patients receiving a standard regimen for R/R MM. For the purpose of our study, ‘elderly’ is broadly defined to include patients aged 65 and above. This range encompasses both the conventional definition of ‘elderly’ and extends to an older subgroup to account for variations in study designs that we came across.

### 2.3. Exclusion Criteria

Studies failing to meet the inclusion criteria were excluded from the analysis, including those that were not comparative or not prospective, had a sample size smaller than 10 participants per arm, had outcomes of interest not reported, or had unclear methodology or data. Additionally, studies not available in English were excluded, as were those published in conference abstracts without full-text availability or were duplicate reports from the same study population.

### 2.4. Risk of Bias Assessment

Two reviewers (CHL and PHC) conducted the assessment of study quality following the methodology and categories outlined in the Cochrane Collaboration Handbook [13]. In instances of disagreement, a group discussion was conducted to reach a consensus. The risk of bias was evaluated in five specific domains, including method of randomization, allocation concealment, blindness, withdrawal or dropout, and adequacy of follow-up. We paid specific attention to baseline imbalances and the funding source when evaluating other issues [14]. The risk of bias graphs were presented using Review Manager software (Version 5.3; The Nordic Cochrane Centre, The Cochrane Collaboration, Copenhagen, Denmark) [15] (Figure S1).

### 2.5. Data Extraction

Two independent reviewers (CHL and CL) evaluated the eligibility of all identified citations. They obtained information on study characteristics such as first author, publication year, treatment options, number of participants, study design, study duration, source of financial support, and patient characteristics, including inclusion criteria and cut-off level of age. They also collected data on sample sizes and details of interventions, including comparisons and outcomes. For each trial, the reviewers evaluated the HR of PFS in the elderly subsets of patients from each selected trial. In cases where the HR of survival curves was unreported [16,17], they calculated it from the graph using the method outlined by Tierney et al. [18]. To minimize the risk of data entry errors, we implemented double data entry and cross-checked the information, and any discrepancies were resolved through group discussions.

### 2.6. Data Synthesis and Statistical Analysis

An NMA was conducted to compare 14 different therapy options for PFS in R/R MM patients [19]. The analysis combined both direct and indirect estimates of the relative treatment effect using the statistical software R and the “Netmeta” package (Version 4.1.2; R Foundation for Statistical Computing, Vienna, Austria) [20]. Dexamethasone was used as the reference treatment, and hazard risk (HR) with 95% confidence intervals (CIs) was calculated using the random-effect frequentist NMA following UK NICE guidance [21]. Statistical significance was defined as a *p*-value below 0.05.

To provide a comprehensive overview of the trials analyzed, a network plot was generated [19]. We assessed the assumption of network transitivity by visually examining tables containing patient characteristics. Incongruencies between direct and indirect effects within a single comparison in the network could result in potential inconsistencies. To identify such inconsistencies, we used a random-effects design-by-treatment interaction model and a node-splitting technique for each comparison [22]. We utilized a forest plot illustrating the estimated summary effects, including confidence intervals, for all comparisons. This approach summarized the relative mean effects, assessed the impact of heterogeneity, and presented predictions for each comparison in a single plot [23].

We utilized SUCRA (surface under the cumulative ranking curve) to determine the probability of a treatment being ranked at a particular position based on the outcome. SUCRA transforms the mean rank into a simple value, which offers a hierarchy of the treatments that considers both the location and variance of all relative treatment effects [24,25,26]. In addition, we adopted a P-score from the concept of SUCRA in the frequentist NMA [27,28]. A higher P-score value, closer to 1, indicates a better rank for the intervention. This analysis allowed us to identify the treatments with the highest probability of being the treatment with the most efficacy in terms of PFS for R/R MM.

## 3. Results

**Figure 1:**
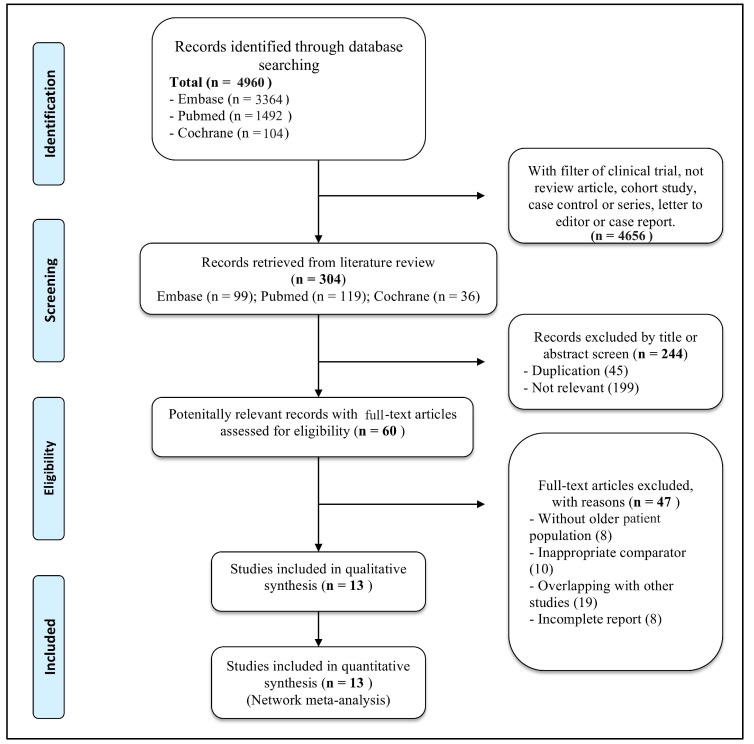
PRISMA flowchart of study selection.

Figure 1 shows the algorithm for this systematic literature review. At the outset, 4966 citations were obtained from the databases. Following the exclusion of non-randomized controlled trials (leaving 304 citations), a screening process based on title and abstract resulted in the exclusion of 244 studies. In the subsequent phase, 60 full texts were reviewed, with 47 exclusions. Ultimately, 13 citations were included for qualitative analysis.

The analysis included 13 RCTs, with a total of 3337 participants. Scheme S1 provides details regarding the search procedure. The 13 RCTs represented 14 treatment arms: bortezomib + dexamethasone (BorDex), dexamethasone (Dex), lenalidomide + dexamethasone (LenDex), carfilzomib + lenalidomide + dexamethasone (CarLenDex), ixazomib + lenalidomide + dexamethasone (IxaLenDex), elotuzumab + lenalidomide + dexamethasone (EloLenDex), daratumumab + lenalidomide + dexamethasone (DaraLenDex), pomalidomide + dexamethasone (PomDex), vorinostat + bortezomib (VorinoBor), panobinostat + bortezomib + dexamethasone (PanoBorDex), carfilzomib + dexamethasone (CarDex), daratumumab + bortezomib + dexamethasone (DaraBorDex), elotuzumab + bortezomib + dexamethasone (EloBorDex), and pegylated liposomal doxorubicin + bortezomib (PegDoxBor).

Table 1 provides a concise overview of the characteristics of the trials included. All studies had subgroup analyses of elderly patients, with 12 trials setting the cut-off level of age at around 65 and one setting the cut-off level at age 75. Eleven trials reported results of PFS, and two trials reported results of time to progression (TTP). 

### 3.1. Network Meta-Analysis

**Figure 2:**
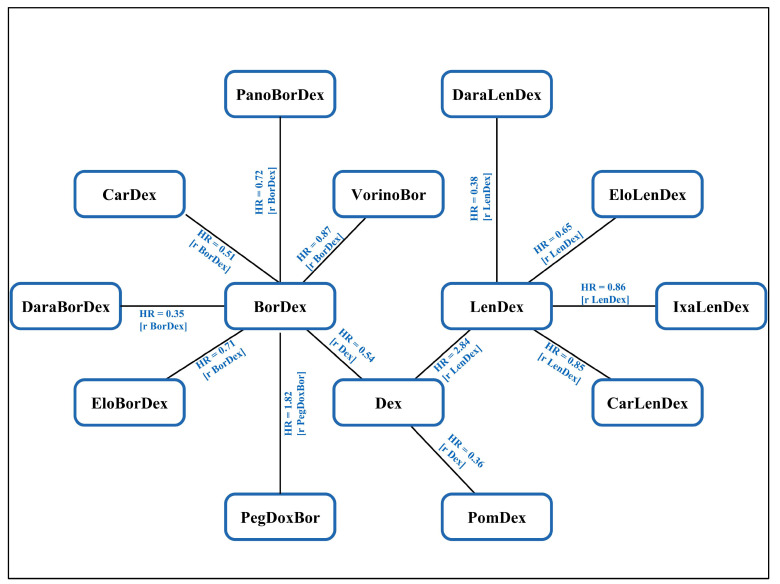
Visual representation illustrating the evidence network utilized in NMA. Directly comparable treatments are linked with a line. Parentheses indicate the reference treatment. Square brackets present the number of directly comparable trials. The HR of PFS is highlighted in blue and the r indicates reference. BorDex, bortezomib + dexamethasone; Dex, dexamethasone; LenDex, lenalidomide + dexamethasone; CarLenDex, carfilzomib + lenalidomide + dexamethasone; IxaLenDex, ixazomib + lenalidomide + dexamethasone; EloLenDex, elotuzumab + lenalidomide + dexamethasone; DaraLenDex, daratumumab + lenalidomide + dexamethasone; PomDex, pomalidomide + dexamethasone; VorinoBor, vorinostat + bortezomib; PanoBorDex, panobinostat + bortezomib + dexamethasone; CarDex, carfilzomib + dexamethasone; DaraBorDex, daratumumab + bortezomib + dexamethasone; EloBorDex, elotuzumab + bortezomib + dexamethasone; PegDoxBor, pegylated liposomal doxorubicin + bortezomib.

Figure 2 describes the integral network of treatment options for R/R MM in elderly patients. Direct comparison results of PFS were described between all treatment options. There were three assumptions adopted for including all trials into a single network [29]: (1) The equal relative efficacy of Bor ([30,31]) versus Dex and BorDex ([32,33,34]) versus Dex. (2) The proxy of TTP in case of lacking PFS data ([31,35]). (3) Identical Bor efficacy via different administration pathways whether intravenous ([30,31,32,33,35]) or subcutaneous ([32,34]).

### 3.2. Progression Free Survival

**Figure 3:**
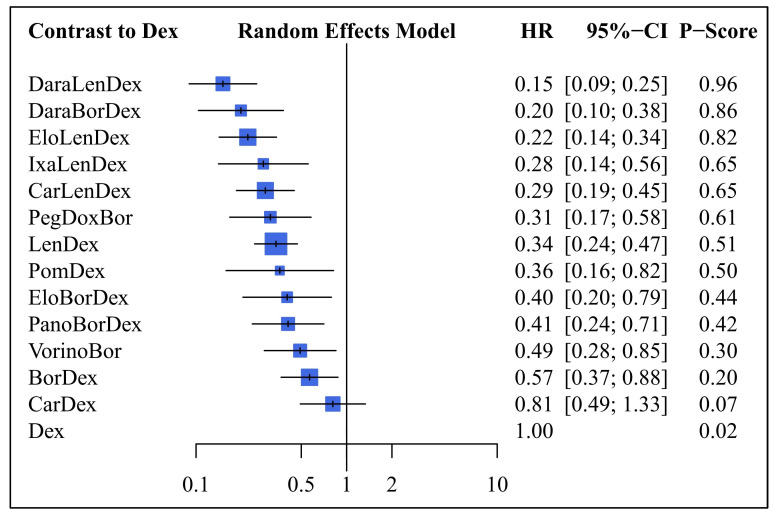
NMA results of treatment efficacy in PFS in R/R MM patients. HR, hazard ratio; P-score indicates the SUCRA (surface under the cumulative ranking curve); CI, confidence interval; BorDex, bortezomib + dexamethasone; Dex, dexamethasone; LenDex, lenalidomide + dexamethasone; CarLenDex, carfilzomib + lenalidomide + dexamethasone; IxaLenDex, ixazomib + lenalidomide + dexamethasone; EloLenDex, elotuzumab + lenalidomide + dexamethasone; DaraLenDex, daratumumab + lenalidomide + dexamethasone; PomDex, pomalidomide + dexamethasone; VorinoBor, vorinostat + bortezomib; PanoBorDex, panobinostat + bortezomib + dexamethasone; CarDex, carfilzomib + dexamethasone; DaraBorDex, daratumumab + bortezomib + dexamethasone; EloBorDex, elotuzumab + bortezomib + dexamethasone; PegDoxBor, pegylated liposomal doxorubicin + bortezomib.

The results of the NMA for PFS are presented in Figure 3. The analysis encompassed a total of 13 studies. Within our network meta-analysis framework, Dex served as the reference treatment, but this does not imply it was the comparator in the majority of the studies. Treatments were prioritized according to their likelihood of being the optimal choice, and their HR with 95% CI are presented.

The analysis showed that the best treatment option for PFS was daratumumab + lenalidomide + dexamethasone (DaraLenDex), with an HR of 0.15 (95% CI: 0.09–0.25) and a probability score (P-score) of 0.96. The second-best treatment options were daratumumab + bortezomib + dexamethasone (DaraBorDex) and elotuzumab + lenalidomide + dexamethasone (EloLenDex), which reported similar rankings.

Overall, 12 out of the 13 treatments were significantly better than Dex, with HR ranging from 0.15 to 0.57. The only treatment combination that did not show a significant treatment effect was carfilzomib + dexamethasone (CarDex). Moreover, 11 treatment combinations demonstrated a risk reduction for progression or death of over 50% compared to dexamethasone (Dex). The most effective treatment option, DaraLenDex, reduced this risk by 85%.

Our analysis revealed no significant evidence of inconsistency between direct and indirect comparisons across the network. It should be noted that we employed a rigorous approach to identifying any potential inconsistencies, using both a random-effects design-by-treatment interaction model and a node-splitting technique for each comparison. Supplementary Figure S1 presents a summary and illustration of the risk of bias evaluation for the included RCTs. The overall quality of the studies included in our analysis met acceptable standards; however, the studies are primarily affected by the risk of bias due to the absence of blinding among participants and unclear blinding of outcome assessors.

## 4. Discussion

### 4.1. Overview and Key Findings

Our study is a NMA of 14 treatment options for patients with R/R MM, comparing their efficacy in terms of PFS. The 13 RCTs enrolled a total of 3337 cases, and all trials had subgroup analyses of elderly patients. The NMA found that daratumumab + lenalidomide + dexamethasone (DaraLenDex) was the best treatment option for R/R MM, with a significant reduction in the risk of progression or death by 85%. DaraBorDex and EloLenDex were ranked as the second-best treatment options. All treatments, except for the combination treatment of carfilzomib + dexamethasone (CarDex), were significantly better than the comparator (dexamethasone). Furthermore, 11 treatment combinations demonstrated a risk reduction for progression or death of over 50% compared to Dex.

### 4.2. Significance and Consistency with Prior Studies

Our study aimed to systematically review and compare therapeutic options available for elderly patients with R/R MM, considering the efficacy of regimens containing novel agents such as PIs, IMiDs, or mAbs. While emerging evidence has reported new combinations of MM treatments, no study has specifically focused on elderly patients. Previous studies have conducted NMA in newly diagnosed MM patients ineligible for transplantation [36] or provided evidence synthesis in R/R MM treatment [29], but none have investigated this specific population. 

A previous NMA conducted by van Beurden-Tan et al. [29] evaluated the treatment outcomes in a general patient population with R/R MM and found that the combination of daratumumab, lenalidomide, and dexamethasone was the best treatment option. This combination had the most favorable HR for PFS and the highest probability of being the best treatment option. Our NMA results were consistent with this study and also showed that the combination of DaraLenDex was the treatment with the highest efficacy for prolonging PFS in the elderly patient population. Despite the difference in the patient population between the studies, both studies reached a similar conclusion regarding the efficacy of the combination treatment of daratumumab, lenalidomide, and dexamethasone for prolonging progression-free survival.

Our study aimed to systematically review and compare all available therapeutic options for R/R MM in elderly patients, with a focus on regimens that included novel agents such as PIs, IMiDs or mAbs. Unlike previous studies, we used HR as an effect measure for PFS, included more recent treatments, and combined all evidence into one single network for R/R MM in aged patients. Although we used a random-effects frequentist NMA and did not investigate heterogeneity due to the limited number of studies [13,36,37,38], our results provide crucial information for healthcare decision-making in the treatment of older patients with R/R MM. However, we did not present an NMA based on OS or response rate outcomes due to a lack of sufficient sub-group analyses from current RCTs. Overall, our study fills an important gap in the literature by providing valuable insights into the optimal treatment options for older patients with R/R MM.

### 4.3. Frailty Considerations and Comprehensive Assessments

Our study accentuates the critical role of frailty assessments in tailoring salvage treatments for elderly patients with refractory/relapsed multiple myeloma, acknowledging the reduced tolerance for intensive regimens compared to younger cohorts. Incorporating tools like the Katz Activities of Daily Living Index and the Charlson Comorbidity Index—as highlighted by Palumbo et al. [9]—enhances the prediction of mortality and toxicity risk beyond chronological age. While younger patients may have options like stem cell transplantation for prolonged survival, such intensive therapies are less suitable for the elderly due to greater procedural risks and comorbidities. Consequently, our findings advocate for less intensive but efficacious regimens, such as triple therapy, and call for comparative studies that integrate comprehensive geriatric assessments to refine treatment protocols for this demographic.

### 4.4. Elderly Criteria and Treatment Optimization Debate

In the realm of MM treatment for elderly patients, the definition of ‘elderly’ and the criteria for treatment optimization have been the subjects of ongoing debate. We chose the age of 65 as our inclusion criterion based on the majority of RCTs that conducted age subgroup analyses. This decision was pragmatic and aimed to maximize the utility of available data. While we recognize the merit in including factors such as frailty and comorbidities, the limited data available for the elderly population posed challenges. Many RCTs do not focus exclusively on elderly patients, making it even more challenging to incorporate comprehensive frailty or comorbidity analyses. However, these factors are undeniably crucial when considering the treatment regime for elderly patients with MM. Their inclusion in future studies would undoubtedly provide a more holistic view of treatment efficacy and safety for this demographic.

### 4.5. Rationale for Dexamethasone as Reference

In our NMA, the choice of dexamethasone as a single agent as the reference merits further elucidation. In the context of NMAs, the selection of a reference treatment is often guided by methodological considerations rather than its prevalent clinical usage. Specifically, employing the lowest efficacy as a reference establishes a baseline that facilitates the differentiation and ranking of the relative efficacy of other treatments. Dexamethasone monotherapy, based on our analysis, offers a theoretically lowest efficacy, hence its selection as a reference point. This approach ensures comparative clarity, as the magnitude of difference in efficacy between other treatments and a ‘lowest efficacy’ reference becomes more discernible. Additionally, even though dexamethasone monotherapy may not be a predominant choice in modern clinical practice, it has historical relevance and is documented in the literature, making it an appropriate comparator. Ultimately, this methodological choice aimed to ensure analytical consistency across the network and to provide a perspective on the relative efficacies of treatments in real-world settings when compared against a recognized baseline.

### 4.6. Underlying Assumptions in NMA

In the realm of NMA, the inherent assumptions and their underlying rationale often dictate the direction, credibility, and interpretability of the findings. For our analysis, one pivotal assumption was the equality of relative efficacy between Bor and Dex, and between BorDex and Dex, based on observations from multiple studies ([30,31,32,33,34]). This assumption was not made arbitrarily but grounded in empirical data. We were compelled to make this decision to bridge the gap between direct and indirect evidence, ensuring that the entirety of our network remains connected and analyzable. By assuming the said equivalences, it allowed us to draw broader conclusions and ensure the network was robust and valid. However, it is crucial to recognize the potential limitations of such assumptions. Equating treatments based on relative efficacy can sometimes oversimplify the true clinical scenario. This is especially true when individual studies might have unique patient populations, methodologies, or outcome measures that can subtly influence results.

### 4.7. Emergence of New Therapeutic Modalities

In recent years, advances in the field of oncology have led to the emergence of innovative therapeutic modalities for multiple myeloma, including chimeric antigen receptor T-cell (CAR-T) therapy, bispecific monoclonal antibodies (MoAbs), and immunoconjugates. These therapies represent a paradigm shift in the treatment landscape, showing promising results in terms of efficacy and safety profiles. However, it is crucial to highlight that while these therapies have demonstrated significant potential in clinical trials, their application to the elderly population remains limited. Elderly patients often present with a range of comorbidities, frailty, and other health considerations that can influence treatment decisions and outcomes. As of our current knowledge cutoff, there are limited data regarding the safety and efficacy of CAR-T, bispecific MoAbs, and immunoconjugates specifically in elderly patients with multiple myeloma. Incorporating these newer treatments into our analysis would certainly be of value. However, the absence of sufficient data and randomized controlled trials focusing on elderly patients restricts our ability to comprehensively evaluate their impact within this specific patient group. As the therapeutic landscape continues to evolve, future research that specifically addresses the utility and safety of these modalities in elderly patients will be paramount.

### 4.8. Study Limitations and Data Sources

While our study offers meaningful insights into the efficacy of various treatments for R/R MM in elderly patients, there are several limitations to consider. Firstly, our reliance on published studies rather than individual patient records could limit the accuracy and comprehensiveness of our findings. Specifically, since a substantial amount of our data was drawn from subgroup analyses of clinical trials, detailed patient characteristics such as median and range of age, performance status, and frailty were not consistently available. This absence might introduce potential biases given the heterogeneity of patient populations and treatment regimens under consideration. As a consequence, comparing studies with analogous patient characteristics was not feasible with the data at hand. Additionally, our analysis is inherently heuristic, providing a snapshot of current evidence which may not capture emerging trends as new research surfaces. Despite these challenges, our study imparts crucial information to guide treatment choices for elderly patients with R/R MM.

### 4.9. Concluding Remarks and Clinical Implications

Our research illuminates optimal treatment paradigms for elderly patients battling refractory/relapsed multiple myeloma. From our NMA, which encapsulates regimens assessed in randomized trials, it appears that a three-drug regimen—merging the lenalidomide-dexamethasone backbone with the anti-MM monoclonal antibody, daratumumab—stands as the most promising treatment in this context. These conclusions underscore the escalating role of immunotherapy in MM management. However, recognizing the potential biases from the heterogeneity of agents and patient populations in our analysis, we advocate for more prospective randomized trials to pinpoint the best sequencing of MM treatments. In the interim, our findings remain pivotal for informed decision-making in clinical settings, pending more direct comparative evidence.

## 5. Conclusions

In conclusion, our network meta-analysis provides valuable information on the efficacy of various treatment options for refractory/relapsed multiple myeloma in elderly patients. Our findings suggest that the three-drug regimen of daratumumab, lenalidomide, and dexamethasone is the treatment with the highest efficacy for prolonging progression-free survival in this patient population.

## Figures and Tables

**Table 1:** Basic characteristics of included randomized trials.

Trial Name/First Author	Number of Patients	Treatment Arm A	Treatment Arm B	Cut-Off Level of Age	Primary Objective
CASTOR (NCT02136134)	241	DaraBorDex	BorDex	65	PFS
ELOQUENT-2 (NCT01239797)	370	EloLenDex	LenDex	65	PFS
MM-003 (NCT01311687)	36	PomDex	Dex	75	PFS
PANORAMA1 (NCT01023308)	323	PanoBorDex	BorDex	65	PFS
POLLUX (NCT02076009)	296	DaraLenDex	LenDex	65	PFS
ENDEAVOR (NCT01568866)	496	CarDex	BorDex	65	PFS
VANTAGE 088 (NCT00773747)	256	VorinoBor	Bor	65	PFS
ASPIRE (NCT01080391)	393	CarLenDex	LenDex	65	PFS
Orlowski (NCT00103506)	250	Bor	PegDoxBor	65	TTP
Jakubowiak (NCT01478048)	85	EloBorDex	BorDex	65	PFS
MM-009 (NCT00056160) MM-010 (NCT00424047)	314	Dex	Dex	65	PFS
APEX (NCT00048230)	245	Bor	Dex	65	TTP
Tourmaline-MM1(NCT01564537)	32	IxaLenDex	LenDex	65	PFS

## Data Availability

The data presented in this study are available on request from the corresponding author.

## Supplementary Materials

The following supporting information can be downloaded at: https://www.mdpi.com/article/10.3390/life13122259/s1. Figure S1. Risk of bias summary. Table S1. PRISMA Checklist. Scheme S1. Search strategies and detailed records.
