# Supplementary material for: Progression-Free Survival Efficacy in Refractory/Relapsed Multiple Myeloma among Elderly Patients: A Systematic Review
Source: Life (Basel). 2023 Nov 27;13(12):2259. doi: 10.3390/life13122259 (PMC10744445; doi:10.3390/life13122259)
Supplement: Supplementary file 1 [file life-13-02259-s001.zip › life-2627633-supplementary.pdf]

life

MDPI

Progression-Free Survival Efficacy in Refractory/Relapsed Multiple Myeloma among Elderly Patients: A Systematic Review

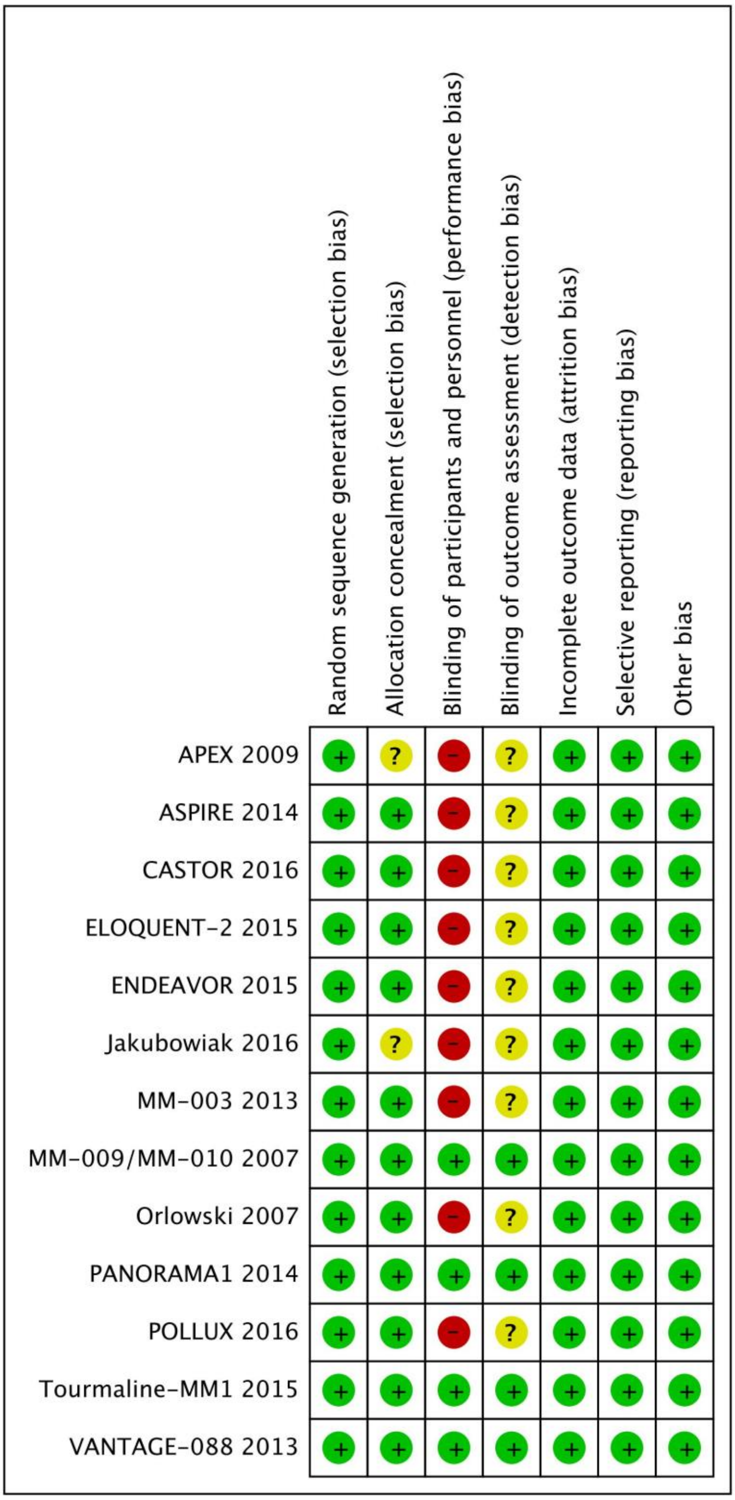

Figure S1. Risk of Bias Summary.

Scheme S1. Search strategies and detailed records.

Relevant text of Population	Relevant text of intervention
1. Multiple myeloma<br>2. Myeloma<br>3. Myeloma cell<br>4. myelom<br>A= #1 or #2 or #3 or #4<br>5. Recurrent<br>6. Refractory<br>7. Relapse<br>B = #5 or #6 or #7<br>8. Progression free survival<br>9. Time to progression<br>10. Survival<br>C = #8 or #9 or #10<br>11. Trial<br>12. Clinical trial/ trials<br>13. Randomized<br>14. Randomization<br>15. Controlled trial/trials<br>16. Randomized controlled trial/trials<br>17. Controlled clinical trial<br>18. RCT<br>19. Persepctive study<br>20. Clinical study<br>21. Clinical article<br>D = #11 or #12 or #13 or #14 or #15 or #16 or #17 or #18 or #19 or #20 or #21	1. Bortezomib<br>2. Lenalidomide<br>3. Thalidomide<br>4. Bendamustine<br>5. Dexamethasone<br>6. Melphalan<br>7. Vincristine<br>8. Cyclophosphamide<br>9. Doxorubicin<br>10. Carmustine<br>11. Prednisone<br>12. Pomalidomide<br>13. Panobinostat<br>14. Carfilzomib<br>15. Daratumumab<br>16. Ixazomib<br>17. Elotuzumab<br>E = 1 or 2 or 3 or 4 or 5 or 6 or 7 or 8 or 9 or 10 or 11 or 12 or 13 or 14 or 15 or 16 or 17<br>*. All search keyword with [Mesh Terms] or [All Fields]

Web sites and uniform resource locator: PUBMED: http://www.ncbi.nlm.nih.gov/pubmed EMBASE: https://www.em-base.com

COCHRANE CENTRAL: https://www.cochrane.com

Search details		
Pubmed data base		
Population		
A & B & C	(multiple myeloma/exp OR multiple myeloma OR myeloma/exp OR myeloma OR myeloma cell/exp OR myeloma cell OR myeloma) AND (recurrent OR refractory OR relapse/exp OR relapse) AND (progression free survival/exp OR progression free survival OR time to progression/exp OR time to progression OR survival/exp OR survival)	
Intervention (including all drugs)		
E	Intervention<br>(bortezomib OR lenalidomide OR thalidomide OR bendamustine OR dexamethasone OR melphalan OR vincristine OR cyclophosphamide OR doxorubicin OR carmustine OR prednisone OR pomalidomide OR panobinostatOR carfilzomib OR daratumumab OR ixazomib OR elotuzumab)	244,203

A & B& C &D & E	PICO (((bortezomib[Title/Abstract] OR lenalidomide[Title/Abstract] OR thalidomide[Title/Abstract] OR bendamustine[Title/Abstract] OR dexamethasone[Title/Abstract] OR melphalan[Title/Abstract] OR vincristine[Title/Abstract] OR cyclophosphamide[Title/Abstract] OR doxorubicin[Title/Abstract] OR carmustine[Title/Abstract] OR prednisone[Title/Abstract] OR pomalidomide[Title/Abstract] OR panobinostat[Title/Abstract] OR carfilzomib[Title/Abstract] OR daratumumab[Title/Abstract] OR ixazomib[Title/Abstract] OR elotuzumab)[Title/Abstract])) AND ((multiple myeloma/exp[Title/Abstract] OR multiple myeloma[Title/Abstract] OR myeloma/exp[Title/Abstract] OR myeloma[Title/Abstract] OR myeloma cell/exp[Title/Abstract] OR myeloma cell[Title/Abstract] OR myeloma)[Title/Abstract] AND (recurrent[Title/Abstract]OR refractory[Title/Abstract] OR relapse/exp[Title/Abstract] OR relapse)[Title/Abstract] AND (progression free survival/exp[Title/Abstract] OR progression free survival[Title/Abstract] OR time to progression/exp[Title/Abstract] OR time to progression[Title/Abstract] OR survival/exp[Title/Abstract] OR survival)[Title/Abstract]) Sort by: Relevance Filters: Clinical Trial; Clinical Study; Randomized Controlled Trial; Clinical Trial, Phase IV; Clinical Trial, Phase III	1492
**Cochrane data base**		
Population		
**A & B & C**	**Population** ((myeloma* OR [mh"multiple myeloma] OR [mh"myeloma cell"]) AND (recurrent OR refractory OR relapse) AND ([progression free survival] OR [time to progression] OR survival));ti,ab,kw	**1,024**
**Intervention (including all drugs)**		
E Intervention	(bortezomib OR lenalidomide OR thalidomide OR bendamustine OR dexamethasone OR	3,243

	melphalan OR vincristine OR cyclophosphamide OR doxorubicin OR carmustine OR prednisone OR pomalidomide OR panobinostat OR carfilzomib OR daratumumab OR ixazomib OR elotuzumab);ti,ab,kw	
A & B& C &D & E	PICO<br>(((myeloma* OR [mh"multiple myeloma] OR [mh"myeloma cell"]) AND (recurrent OR refractory OR relapse) AND ([progression free survival] OR [time to progression] OR survival));ti,ab,kw) AND ((bortezomib OR lenalidomide OR thalidomide OR bendamustine OR dexamethasone OR melphalan OR vincristine OR cyclophosphamide OR doxorubicin OR carmustine OR prednisone OR pomalidomide OR panobinostat OR carfilzomib OR daratumumab OR ixazomib OR elotuzumab);ti,ab,kw) AND ((clinical trial OR randomized controlled trial OR perspective OR clinical study OR clinical article OR controlled study)	
Embase data bass		
Population & Outcome		
A & B& C	('multiple myeloma'/exp OR 'multiple myeloma' OR 'myeloma'/exp OR 'myeloma' 6,358 OR 'myeloma cell'/exp OR 'myeloma cell' OR myelom) AND (recurrent OR<br>refractory OR 'relapse'/exp OR 'relapse') AND ('progression free survival'/exp OR 'progression free survival' OR 'time to progression'/exp OR 'time to progression' OR 'survival'/exp OR 'survival'	104
Intervention (including all drugs)		

E	Intervention<br>'bortezomib'/exp OR 'bortezomib' OR 'lenalidomide'/exp OR 'lenalidomide' OR 'thalidomide'/exp OR 'thalidomide' OR 'bendamustine'/exp OR 'bendamustine' OR 'dexamethasone'/exp OR 'dexamethasone' OR 'melphalan'/exp OR 'melphalan' OR 'vincristine'/exp OR 'vincristine' OR 'cyclophosphamide'/exp OR 'cyclophosphamide' OR 'doxorubicin'/exp OR 'doxorubicin' OR 'carmustine'/exp OR 'carmustine' OR 'prednisone'/exp OR 'prednisone' OR 'pomalidomide'/exp OR 'pomalidomide' OR 'panobinostat'/exp OR 'panobinostat' OR 'carfilzomib'/exp OR 'carfilzomib' OR 'daratumumab'/exp OR 'daratumumab' OR 'ixazomib'/exp OR 'ixazomib' OR 'elotuzumab'/exp OR 'elotuzumab'	598,883
A & B& C &D & E	PICO<br>('multiple myeloma'/exp OR 'multiple myeloma' OR 'myeloma'/exp OR 'myeloma' OR 'myeloma cell'/exp OR 'myeloma cell' OR myelom) AND (recurrent OR refractory OR 'relapse'/exp OR 'relapse') AND ('bortezomib'/exp OR 'bortezomib' OR 'lenalidomide'/exp OR 'lenalidomide' OR 'thalidomide'/exp OR 'thalidomide' OR 'bendamustine'/exp OR 'bendamustine' OR 'dexamethasone'/exp OR 'dexamethasone' OR 'melphalan'/exp OR 'melphalan' OR 'vincristine'/exp OR 'vincristine' OR 'cyclophosphamide'/exp OR 'cyclophosphamide' OR 'doxorubicin'/exp OR 'doxorubicin' OR 'carmustine'/exp OR 'carmustine' OR 'prednisone'/exp OR 'prednisone' OR 'pomalidomide'/exp OR 'pomalidomide' OR 'panobinostat'/exp OR 'panobinostat' OR 'carfilzomib'/exp OR 'carfilzomib' OR 'daratumumab'/exp OR 'daratumumab' OR 'ixazomib'/exp OR 'ixazomib' OR 'elotuzumab'/exp OR 'elotuzumab') AND ('progression free survival'/exp OR 'progression free survival' OR	3,364

	'time to progression'/exp OR 'time to progression' OR 'survival'/exp OR 'survival') AND ('clinical trial'/exp OR 'clinical trial' OR 'randomized controlled trial'/exp OR 'randomized controlled trial' OR 'perspective'/exp OR 'perspective' OR 'clinical study'/exp OR 'clinical study' OR 'clinical article'/exp OR 'clinical article' OR 'controlled study'/exp OR 'controlled study')	
