# Supplementary material for: Progression-Free Survival Efficacy in Refractory/Relapsed Multiple Myeloma among Elderly Patients: A Systematic Review
Source: Life (Basel). 2023 Nov 27;13(12):2259. doi: 10.3390/life13122259 (PMC10744445; doi:10.3390/life13122259)
Supplement: Supplementary file 1 [file life-13-02259-s001.zip › Supplemental Table PRISMA Checklist.pdf]

Supplemental Table PRISMA Checklist

Section/ Topic	Item	Checklist Item	Reported on Page #
TITLE			
Title	1	Identify the report as a systematic review *incorporating a network meta-analysis (or related form of meta-analysis).*	1
ABSTRACT			
Structured summary	2	Provide a structured summary including, as applicable:<br>Background: main objectives<br>Methods: data sources; study eligibility criteria, participants, and interventions; study appraisal; and *synthesis methods, such as network meta-analysis.*<br>Results: number of studies and participants identified; summary estimates with corresponding confidence/credible intervals; *treatment rankings may also be discussed. Authors may choose to summarize pairwise comparisons against a chosen treatment included in their analyses for brevity.*<br>Discussion/Conclusions: limitations; conclusions and implications of findings.<br>Other: primary source of funding; systematic review registration number with registry name.	1
INTRODUCTION			
Rationale	3	Describe the rationale for the review in the context of what is already known, *including mention of why a network meta-analysis has been conducted.*	1
Objectives	4	Provide an explicit statement of questions being addressed, with reference to participants, interventions, comparisons, outcomes, and study design (PICOS).	2
METHODS			
Protocol and registration	5	Indicate whether a review protocol exists and if and where it can be accessed (e.g., Web address); and, if available, provide registration information, including registration number.	2
Eligibility criteria	6	Specify study characteristics (e.g., PICOS, length of follow-up) and report characteristics (e.g., years considered, language, publication status) used as criteria for eligibility, giving rationale. *Clearly describe eligible treatments included in the treatment network, and note whether any have been clustered or merged into the same node (with justification).*	3
Information sources	7	Describe all information sources (e.g., databases with dates of coverage, contact with study authors to identify additional studies) in the search and date last searched.	3

Search	8	Present full electronic search strategy for at least one database, including any limits used, such that it could be repeated.	Supplemental Table
Study selection	9	State the process for selecting studies (i.e., screening, eligibilit y, included in systematic review, and, if applicable, included i n the meta-analysis).	3
Data collection process	10	Describe method of data extraction from reports (e.g., piloted f orms, independently, in duplicate) and any processes for obtai ning and confirming data from investigators.	3
Data items	11	List and define all variables for which data were sought (e.g., PICOS, funding sources) and any assumptions and simplificati ons made.	3
Geometry of the network	S1	Describe methods used to explore the geometry of the treatme nt network under study and potential biases related to it. This s hould include how the evidence base has been graphically sum marized for presentation, and what characteristics were compil ed and used to describe the evidence base to readers.	4
Risk of bias wit hin individual st udies	12	Describe methods used for assessing risk of bias of individual studies (including specification of whether this was done at th e study or outcome level), and how this information is to be us ed in any data synthesis.	4
Summary meas ures	13	State the principal summary measures (e.g., risk ratio, differen ce in means). *Also describe the use of additional summary me asures assessed, such as treatment rankings and surface under t he cumulative ranking curve (SUCRA) values, as well as modi fied approaches used to present summary findings from meta-analyses.*	4
Planned method s of analysis	14	Describe the methods of handling data and combining results of studies for each network meta-analysis. This should includ e, but not be limited to:<br>*Handling of multi-arm trials;*<br>*Selection of variance structure;*<br>*Selection of prior distributions in Bayesian analyses; and*<br>*Assessment of model fit.*	4
Assessment of I nconsistency	S2	Describe the statistical methods used to evaluate the agreemen t of direct and indirect evidence in the treatment network(s) st udied. Describe efforts taken to address its presence when fou nd.	4
Risk of bias acr oss studies	15	Specify any assessment of risk of bias that may affect the cum ulative evidence (e.g., publication bias, selective reporting wit hin studies).	4

Additional analyses	16	Describe methods of additional analyses if done, indicating which were pre-specified. This may include, but not be limited to, the following:<br>Sensitivity or subgroup analyses;<br>Meta-regression analyses;<br>*Alternative formulations of the treatment network; and Use of alternative prior distributions for Bayesian analyses (if applicable).*	-
RESULTS†			
Study selection	17	Give numbers of studies screened, assessed for eligibility, and included in the review, with reasons for exclusions at each stage, ideally with a flow diagram.	4
Presentation of network structure	S3	Provide a network graph of the included studies to enable visualization of the geometry of the treatment network.	Figure 2
Summary of network geometry	S4	Provide a brief overview of characteristics of the treatment network. This may include commentary on the abundance of trials and randomized patients for the different interventions and pairwise comparisons in the network, gaps of evidence in the treatment network, and potential biases reflected by the network structure.	Table 1
Study characteristics	18	For each study, present characteristics for which data were extracted (e.g., study size, PICOS, follow-up period) and provide the citations.	8
Risk of bias within studies	19	Present data on risk of bias of each study and, if available, any outcome level assessment.	Supplemental Figure
Results of individual studies	20	For all outcomes considered (benefits or harms), present, for each study: 1) simple summary data for each intervention group, and 2) effect estimates and confidence intervals. *Modified approaches may be needed to deal with information from larger networks.*	Supplemental Figure
Synthesis of results	21	Present results of each meta-analysis done, including confidence/credible intervals. *In larger networks, authors may focus on comparisons versus a particular comparator (e.g. placebo or standard care), with full findings presented in an appendix. League tables and forest plots may be considered to summarize pairwise comparisons.* If additional summary measures were explored (such as treatment rankings), these should also be presented.	Figure 3
Exploration for inconsistency	S5	Describe results from investigations of inconsistency. This may include such information as measures of model fit to compare consistency and inconsistency models, *P* values from statistical tests, or summary of inconsistency estimates from different parts of the treatment network.	8

Risk of bias acr oss studies	22	Present results of any assessment of risk of bias across studies for the evidence base being studied.	8
Results of additi onal analyses	23	Give results of additional analyses, if done (e.g., sensitivity or subgroup analyses, meta-regression analyses, *alternative netw ork geometries studied, alternative choice of prior distributions for Bayesian analyses,* and so forth).	-
DISCUSSION			
Summary of evi dence	24	Summarize the main findings, including the strength of eviden ce for each main outcome; consider their relevance to key gro ups (e.g., healthcare providers, users, and policy-makers).	9
Limitations	25	Discuss limitations at study and outcome level (e.g., risk of bi as), and at review level (e.g., incomplete retrieval of identified research, reporting bias). *Comment on the validity of the assu mptions, such as transitivity and consistency. Comment on an y concerns regarding network geometry (e.g., avoidance of cer tain comparisons).*	11
Conclusions	26	Provide a general interpretation of the results in the context of other evidence, and implications for future research.	11
FUNDING			
Funding	27	Describe sources of funding for the systematic review and oth er support (e.g., supply of data); role of funders for the system atic review. This should also include information regarding wh ether funding has been received from manufacturers of treatm ents in the network and/or whether some of the authors are co ntent experts with professional conflicts of interest that could affect use of treatments in the network.	12

PICOS = population, intervention, comparators, outcomes, study design.
* Text in italics indicates wording specific to reporting of network meta-analyses that has been added to gui dance from the PRISMA statement.
